# Downregulation of KRAB zinc finger proteins in 5-fluorouracil resistant colorectal cancer cells

**DOI:** 10.1186/s12885-022-09417-3

**Published:** 2022-04-04

**Authors:** Anaïs Chauvin, Danny Bergeron, Jean Vencic, Dominique Lévesque, Benoit Paquette, Michelle S. Scott, François-Michel Boisvert

**Affiliations:** 1 Department of Immunology and Cell Biology, Université de Sherbrooke, 3201 Jean-Mignault, Sherbrooke, Québec, J1E 4K8 Canada; 2 Department of Biochemistry and Functional Genomics, Université de Sherbrooke, 3201 Jean-Mignault, Sherbrooke, Québec, J1E 4K8 Canada; 3 Department of Nuclear Medicine and Radiobiology, Université de Sherbrooke, 3201 Jean-Mignault, Sherbrooke, Québec, J1E 4K8 Canada

**Keywords:** Colorectal cancer, Radiation therapy, Chemotherapy, 5-fluorouracil, DNA damage, Drug resistance mechanisms, Cancer omics

## Abstract

Radio-chemotherapy with 5-flu orouracil (5-FU) is the standard of care treatment for patients with colorectal cancer, but it is only effective for a third of them. Despite our understanding of the mechanism of action of 5-FU, drug resistance remains a significant limitation to the clinical use of 5-FU, as both intrinsic and acquired chemoresistance represents the major obstacles for the success of 5-FU-based chemotherapy. In order to identify the mechanism of acquired resistance, 5-FU chemoresistance was induced in CRC cell lines by passaging cells with increasing concentrations of 5-FU. To study global molecular changes, quantitative proteomics and transcriptomics analyses were performed on these cell lines, comparing the resistant cells as well as the effect of chemo and radiotherapy. Interestingly, a very high proportion of downregulated genes were annotated as transcription factors coding for Krüppel-associated box (KRAB) domain-containing zinc-finger proteins (KZFPs), the largest family of transcriptional repressors. Among nearly 350 KRAB-ZFPs, almost a quarter were downregulated after the induction of a 5-FU-resistance including a common one between the three CRC cell lines, ZNF649, whose role is still unknown. To confirm the observations of the proteomic and transcriptomic approaches, the abundance of 20 different KZFPs and control mRNAs was validated by RT-qPCR. In fact, several KZFPs were no longer detectable using qPCR in cell lines resistant to 5-FU, and the KZFPs that were downregulated only in one or two cell lines showed similar pattern of expression as measured by the omics approaches. This proteomic, transcriptomic and genomic analysis of intrinsic and acquired resistance highlights a possible new mechanism involved in the cellular adaptation to 5-FU and therefore identifies potential new therapeutic targets to overcome this resistance.

**Supplementary Information:**

The online version contains supplementary material available at 10.1186/s12885-022-09417-3.

## Introduction

Colorectal cancer (CRC) is the second deadliest cancer worldwide after lung cancer [1], and is therefore a highly studied cancer and many classifications have emerged taking into account the histologic, clinical, pharmacological or even genetic characteristics [2–11]. Anti-pyrimidic 5-fluorouracil (5-FU) is the current standard of therapy to treat CRC, but resistanc     e to fluoropyrimidines is quite common in tumours that recur. 5-FU is an anti-pyrimidic antimetabolite used as a chemotherapy treatment since 1957 [12], and has been one of the most commonly used drugs in adjuvant therapies by inhibition of cancer cell growth and initiation of apoptosis [13]. The anti-pyrimidic metabolite 5-FU is an analogue of uracil with a fluorine atom at position C5 instead of hydrogen [14]. It is widely used for the treatment of colorectal and breast cancers, as well as cancer of the aerodigestive tract, and is still today considered the treatment of reference [15]. 5-FU is often used in combination with other chemotherapeutic agents to increase its potential of action such as leucovorin [16, 17], or with oxaliplatin [18] and irinotecan [19].

5-FU exerts its effect by inducing DNA damage then cell death by three different mechanisms [20]. These cytotoxic effects are the results of different metabolites produced in cells from the conversion of 5-FU into fluorodeoxyuridine monophosphate (FdUMP), fluorouridine triphosphate (FUTP) and fluorodeoxyuridine triphosphate (FdUTP). FdUMP forms a stable complex with thymidylate synthase (TS) and prevents its activity, resulting in depletion of dTMP as it is the only source of *de novo* thymidyl which is necessary for DNA replication and repair [20–22]. Leucovorin is used in combination with 5-FU, which increases the stability of the FdUMP-TS complex [23]. 5-FU is also incorporated into RNA in the form of FUTP in the place of uracil, interfering with the maturation of transcripts [24–28]. It not only inhibits the processing of pre-rRNA into mature rRNA [28–34], but also disrupts post-transcriptional modification of tRNAs [28, 29, 35] as well as assembly and activity of snRNA/protein complexes, thereby inhibiting splicing of pre-mRNA [36]. Moreover, 5-FU can also inhibit polyadenylation of mRNA, therefore disrupting mRNA stability and translation [32, 33]. dUTP and the 5-FU metabolite FdUTP can be misincorporated into DNA [20]. Repair of uracil and 5-FU-containing DNA by the nucleotide excision repair enzyme uracil-DNA-glycosylase (UDG) is futile in the presence of high FdUTP/dTTP ratios and only results in further false-nucleotide incorporation [37, 38]. These cycles of misincorporation, excision and repair eventually lead to accumulation of DNA strand breaks and cell death [39].

Despite our understanding of the mechanism of action of 5-FU, drug resistance remains a significant limitation to the clinical use of 5-FU, as both intrinsic and acquired chemoresistance represents the major obstacles for the success of 5-FU-based chemotherapy [20, 40]. Intrinsic resistance is the innate ability of cells to resist the activity of a particular treatment, and explains the differences in sensitivity of tumors and cancer cells prior to receiving any treatment [41, 42]. However, these resistances are generally below the concentrations typically used during the treatment, and explain the differences in side effects or the extent of the efficacy of the treatment [43]. For example, high thymidylate synthase protein expression is a major 5-FU tolerance factor [44], but high TS expression does not account for non-responding tumors in patients with CRC treated with 5-FU [45]. 5-FU sensitivity is also influenced by several other factors, such as the levels of dihydropyrimidine dehydrogenase [46, 47], the genetic status of TP53 [48], Bcl-2 mediated apoptosis and DNA mismatch repair genes [49]. Additional gene expression data suggest that altered regulation of nucleotide metabolism, amino acid metabolism, cytoskeleton organization, transport, and oxygen metabolism may underlie the intrinsic resistance to 5-FU observed in cell lines [50–52]. For example, specific ABC transporters such as ABCC5 and ABCC11 are also involved in resistance to 5-FU by increasing the efflux of 5-FU out of the cell [47, 51]. In contrast, acquired resistance occurs when the cells develop the ability to resist the activity of a particular treatment to which it was previously susceptible, at therapeutic concentrations [43]. Different studies identified genes associated with acquired 5-FU resistance which explain tolerance to varying concentration of 5-FU, but do not explain how cells become resistant to high concentrations of 5-FU. As such, the precise molecular mechanisms of 5-FU chemoresistance in cancer cells and patients are still largely unknown.

Several comparisons exist between genomic characteristics of CRC and the response to 5-FU. For example, CRC with a defect in mismatch repair (MMR) genes due to microsatellite instabilities (MSI+) would be more resistant to 5-FU because of their inability to recognize and/or react to the incorporation of 5-FU in DNA, but it cannot alone explain the resistance to 5-FU [53, 54]. In their classification, Salazar *et al.* divide CRC into three groups according to their phenotype: A, proliferative, B, epithelial and C, mesenchymal [11]. Later, they bring a pharmacological dimension to subtypes B and C: these subtypes would be predictive of sensitivity and resistance to 5-FU chemotherapy, respectively [10]. Nevertheless, the molecular mechanisms of resistance to chemotherapy, especially to 5-FU and to radiation therapy, are still under investigation in order to find solutions to overcome this resistance. Understanding the mechanisms involved in the resistance to 5-FU could be useful not only to identify potential new druggable targets, but also to predict the clinical response to standard adjuvant chemotherapy [55, 56].

In this study, we investigated intrinsic and acquired resistance to chemotherapy and radiation therapy in CRC cell lines to highlight molecular mechanisms potentially involved in this resistance, and identified proteins from the Krüppel-associated box (KRAB) domain containing zinc-finger proteins (KZFPs) as downregulated in colorectal cancer cell lines that are resistant to 5-FU.

## Materials and methods

### Colorectal cancer cell lines

CRC cell lines were purchased from the American Type Culture Collection (ATCC). Caco-2/15 (ATCC® HTB-37), DLD-1 (ATCC® CCL-221), HCT-116 (ATCC® CCL-247), HT-29 (ATCC® HTB-38), SW480 (ATCC® CCL-228) and SW620 (ATCC® CCL-227) cell lines were grown as adherent cells in Dulbecco’s modified eagle medium (DMEM) supplemented with 10% fetal bovine serum (FBS), 1% penicillin/streptomycin and 1% amphotericin B, at 37 °C with 5% CO_2_. The RRIDs of each cell line are listed in the Key Resources Table. The genomic identity of each cell line is described in Table 1. Corresponding 5-FU-resistant CRC cell lines were obtained after protocol described below and were cultured in DMEM medium supplemented similarly +50 μM 5-FU (#F6627) and at the same temperature and growth conditions.

**Table 1:** Colorectal cancer cell lines used for the study

CRC cell line	Caco-2/15	DLD-1	HCT-116	HT-29	SW480	SW620
**TNM stage**	?	III	?	II/III	II	III
**Genetic profile**						
**CIN**	+	–	–	+	+	+
**MSI**	–	+	+	–	–	–
**CIMP**	–	+	+	+	–	–
**CMS and proteomic subtypes**						
**CMS subtype**	2	N/A	1	3	N/A	N/A
**Proteomic subtype**	A	B	B	D	E	B
**Mutations**						
***KRAS***	–	G13D	G13D	–	G12V	G12V
***BRAF***	–	–	–	V600E	–	–
***PIK3CA***	–	E545K; D549N	H1047R	P449T	–	–
***TP53***	E204X	S241F	–	R273H	R273H; P309S	R273H; P309S
**Radio- and chemo-sensitivities**						
**Radio-sensitivity (LD50)**	4.3 Gy	2.9 Gy	1.7 Gy	4.6 Gy	2.4 Gy	2.7 Gy
**5-FU-sensitivity (IC**_**50**_**)**	2.1 μM	9.3 μM	4.5 μM	2.7 μM	2.4 μM	6.7 μM

**Fig. 1:**
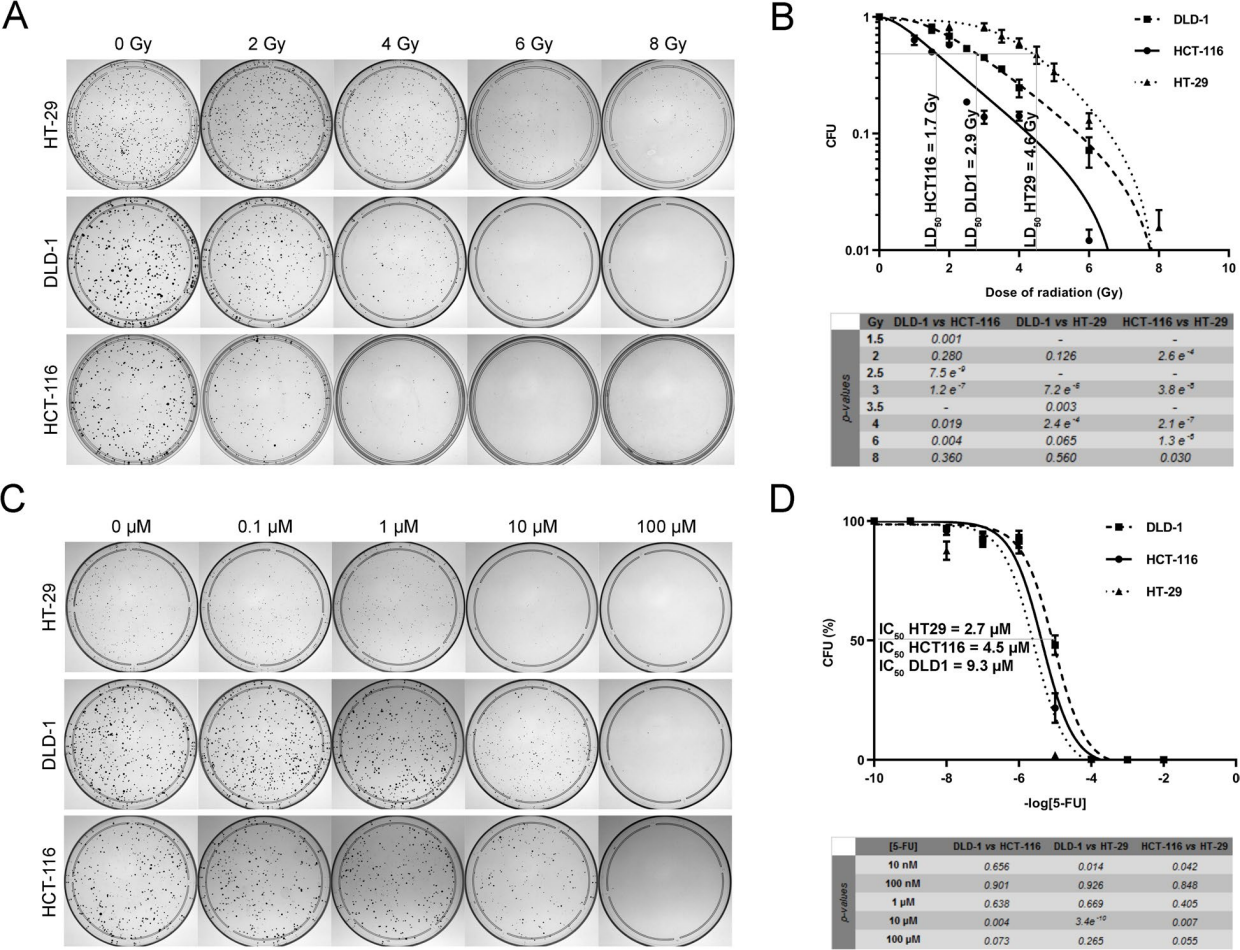
Colorectal cancer cell lines show different intrinsic radio- and chemo-sensitivities. **A** CFU in response to radiations. For each cell line, 1000 cells were seeded at day 0 and irradiated with 2, 4, 6 or 8 Gy at D + 1. CFU from DLD-1 and HCT-116 or HT-29 were counted after an 8-days or 12-days incubation, respectively. **B** Radiation sensitivity of CRC cell lines. LD_50_ values are indicated on the graph for the three cell lines (three parallel samples in three independent experiments). Student’s t-test was used to compare the results obtained for each radiation dose and p-values were indicated in the Table. **C** CFU in response to 5-FU. From each cell line, 1000 cells were seeded at day 0 and treated for 24 h with or without 5-FU (0.1, 1, 10 or 100 μM) at D + 1. CFU from DLD-1 and HCT-116 or HT-29 were counted after an 8-days or 12-days incubation, respectively. Colonies were counted using ImageJ [73]. **D** 5-FU sensitivity of CRC cell lines. IC_50_ values are indicated on the graph for the three cell lines (three parallel samples in three independent experiments). Student’s t-test was used to compare the results obtained for each 5-FU dose and p-values were indicated in the Table. 5-FU: 5-fluorouracil; CFU: colony forming unit; CRC: colorectal cancer; Gy: Gray; LD_50_: half maximal lethal dose; IC_50_: half-maximal inhibitory concentration

The table lists the TNM stages of the patients which these lines come from. Genetic profiles CIN, MSI and CIMP are indicated (+) as well as the CMS and proteomic subtypes [2, 57, 58], the associated mutations [59–63] and the LD50 and IC_50_ previously measured (Fig. 1, Fig. S1). *TNM* tumor, nodes, metastasis, *CIN* chromosomal instability, *MSI* microsatellite instability, *CIMP* CpG island methylator phenotype, *CMS* consensus molecular subtype, *KRAS* V-Ki-ras2 Kirsten rat sarcoma viral oncogene homolog, *BRAF* v-Raf murine sarcoma viral oncogene homolog B1, *PIK3CA* phosphatidylinositol 4,5-bisphosphate 3-kinase catalytic subunit alpha isoform, *TP53* cellular tumor antigen p53

### 5-FU-chemoresistance induction and cell viability assays

To establish the acquired resistant cell lines, DLD-1, HCT-116 and HT-29 were grown in medium supplemented with increasing concentrations of 5-FU for 8 months. The cells underwent three passages in increasing concentrations of 5-FU: 2 μM then 5 μM then increasing by 5 μM at each step, up to 50 μM. The resistant cells are maintained in a medium supplemented with 50 μM 5-FU. For the cell viability assays, 20,000 sensitive and resistant cells were grown in 6-well plate (D0) for two days. They were treated with either 10 μM uracil (control, #U1128), or 10 μM 5-FU or 50 μM 5-FU at D + 2. There were counted using a hemocytometer at D + 2, D + 4 and D + 7 (three parallel samples in three independent experiments for each point for each cell line). Dose-response curves were obtained with GraphPad Prism 8.1.2 for Windows (GraphPad Software, San Diego, California USA, www.graphpad.com).

### DNA damage assays

A time course was carried out to assess the presence of DNA damage. Four experimental conditions were tested: (1) control, (2) chemotherapy alone, (3) radiation therapy alone and (4) chemotherapy combined with radiation therapy. 500,000 DLD-1, HCT-116 and HT-29 cells were grown in 100 mm Petri dish for 48 h. Cells were either (1) treated with uracil 10 μM for 1 and 24 h, or (2) treated with 5-FU 10 μM for 1, 4 and 24 h, or (3) irradiated with the corresponding LD50 using the irradiator X-RAD 225 XL (Precision X-ray) and lysed after 1, 4 and 24 h post-irradiation, or (4) treated with 5-FU 10 μM for 24 h, irradiated with the corresponding LD50 and lysed after 1, 4 and 24 h post-irradiation.

#### Proteomics

##### Experimental conditions

Three experimental conditions were tested: 1) control, 2) chemotherapy alone and 3) chemotherapy combined with radiotherapy. 250,000 DLD-1, HCT-116 and HT-29 cells were grown in 100 mm Petri dish for 72 h. They were treated with either 10 μM uracil (condition 1) or 10 μM 5-FU (condition 2) for 24 h. Medium was changed and cells were irradiated with corresponding LD50 using an X-ray irradiator (X-RAD 225 XL, Precision X-Ray) (condition 3). The device was operated as per manufacturer’s settings for cultured cells (225 kV, 2 mm aluminum filter at 13.3 mA). The same conditions with the corresponding resistant cell lines were conditions 4 to 6. All the experimental conditions were described in Fig. S2 and Table S1.

##### Preparation of whole-cell protein extracts

The cells were washed three times in cold PBS 1X, harvested in cold PBS 1X and centrifuged at 500 *x g* at 4 °C for 5 min. The pellets were lysed in 8 M urea 10 mM HEPES pH 8.0 and centrifuged at 16000 *x g* at 4 °C for 10 min. The amounts of protein in the supernatants were measured with Thermo Scientific™ Bicinchoninic acid (BCA) protein assay kit (#23225) and an amount of 50 μg of proteins was kept for each sample. The volumes were adjusted to 100 μL with 100 mM TEAB dissolution buffer (triethyl ammonium bicarbonate #90114).

##### Reduction and alkylation

Five μL of 200 mM TCEP reducing agent (Bond-Breaker™ Tris-(2-carboxyethyl)-phosphine solution #77720) were added to each sample, which was then incubated at 55 °C for 1 h. Five μL of 375 μM CAA alkylated agent (chloroacetamide) were added to each sample, which was then incubated at room temperature in the dark for 30 min. Six volumes of chilled acetone was added, and proteins were precipitated at −20 °C for at least 4 h. The samples were centrifuged at 8000 *x g* at 4 °C for 10 min, and then the pellets were dried.

##### Protein digestion

The pellets were resuspended in 100 μL of 50 mM TEAB. 1.25 μg of 12.5 ng/ml Trypsin Gold (Promega #V5280) modified in 50 mM acetic acid was added then the samples were digested at 37 °C under agitation overnight.

##### Peptide labelling, purification and desalting

Each TMTsixplex label reagent (#90066) was equilibrated at room temperature, dissolved in 41 μl anhydrous acetonitrile and added in a 50 μg-peptide sample (more details in Table S1). The samples were incubated at room temperature for 1 h. Four μl of 5% hydroxylamine quenching reagent (#90115) were added to the samples, which were then incubated at room temperature for 15 min. The six conditions were pooled and peptides were purified and desalted as described here [64].

##### High performance liquid chromatography separation coupled to mass spectrometry (HPLC-MS/MS)

Trypsin-digested TMT-labelled peptides were separated by HPLC-MS/MS. Two-hundreds nanograms of digested peptides were injected into an HPLC (nanoElute, Bruker Daltonics) and loaded onto a trap column with a constant flow of 4 μL/min (Acclaim PepMap100 C18 column, 0.3 mm id x 5 mm, Dionex Corporation). Peptides were then eluted onto an analytical C18 Column (1.9 μm beads size, 75 μm x 25 cm, PepSep) over a 2-h gradient of acetonitrile (5–37%) in 0.1% formic acid (FA) at 500 nL/min while being injected into a TimsTOF Pro Mass Spectrometer coupled with a Captive Spray nano-electrospray source (Bruker Daltonics). Data were acquired using data-dependent auto-MS/MS with a 100–1700 m/z mass range, with PASEF enabled with a number of PASEF scans set at 10 (1.27 s duty cycle) and a dynamic exclusion of 0.4 min, m/z dependent isolation window and collision energy of 42.0 eV. The target intensity was set to 20,000, with an intensity threshold of 2500.

##### Quantification and bioinformatics analysis

Feature extraction, database searching, and quantitation were performed with the MaxQuant version 1.5.2.8 software [65] and the Uniprot human protein database (version 10/04/2018, 20,368 entries). The following settings were used for the MaxQuant analysis: fixed modifications were carbamidomethylation on cysteine; enzyme was trypsin (K/R not before P); maximum 2 missed cleavages per peptides were allowed; variable modifications were oxidation (M), acetylation (N-terminal), phosphorylation (STY) and carbamylation (K/N-terminal). Quantification was performed through TMT-6plex (CID/HCD) with a mass tolerance of 0.2 Da, an FDR threshold of 5% and reporter ion type was MS2. For each protein, the sum of the intensities between the replicates were realized and the ratios between the six conditions were calculated, then the z-scores (confidence interval of 5%).

#### Transcriptomics

##### Experimental conditions

The same experimental conditions as “Proteomics – Experimental conditions” section were used for transcriptomics experiments.

##### Preparation of total RNA extracts

Total RNA from the six conditions for the three CRC cell lines in triplicate was extracted using RNeasy RNA isolation kit (#74104). The RNA concentrations were measured by NanoDrop and RNA qualities were evaluated on 1.2% agarose gel. The 54 samples were sent to McGill University and Genome Quebec Innovation Center.

##### Preparation of libraries and sequencing

Libraries were generated from 250 ng of total RNA. mRNA enrichment was performed using the NEB Next® Poly(A) mRNA Magnetic Isolation Module (New England Biolabs, Ipswich, USA). The cDNA synthesis was performed using NEBNext RNA® First Strand Synthesis and NEBNext® Ultra™ II Directional RNA Second Strand Synthesis modules (New England Biolabs, Ipswich, USA). The final steps were performed using the NEBNext® Ultra™ II DNA Library Prep Kit for Illumina® (New England Biolabs, Ipswich, USA). PCR adapters and primers were obtained from New England Biolabs. The libraries were quantified by Kapa Illumina GA with Revised Primers-SYBR Universal Fast Kit (Kapa Biosystems, Wilmington, USA). The average size of the RNA fragments was determined using the LabChip GX instrument (PerkinElmer, Waltham, USA). Sequencing of the libraries was performed using NovaSeq 6000 (Illumina, San Diego, USA) using an S4 PE100 protocol.

##### Sequence alignment

RNA-seq analysis was carried out as described previously [66]. Briefly, raw data, obtained from the McGill Genome Centre in BAM format, were reversed to FASTQ format using the Picard 2.21.6 tools (https://broadinstitute.github.io/picard/). RNA read quality was assessed using FastQC 0.11.8 (30254741) and the Trimmomatic 0.36 tool (24695404) was used to preprocess the data [67]. The reads were aligned, and transcripts were quantified using Kallisto 0.44.0 software [68]. The human genome GRCh38.p13 and annotation from ENSEMBL (Homo sapiens version 98) were used to create the transcriptome annotation. Gene counts and gene TPM were obtained by summing the corresponding value of each transcript of a gene. The differential expression was performed using the DESeq2 1.14.1 R package [69].

#### QUANTIFICATION AND STATISTICAL ANALYSIS

All experiments were performed on biological replicates. Sample sizes for each experiment are reported in the corresponding figure legends and methods. Statistically significant differences between control and experimental groups were determined using a test which is reported in the corresponding figure legends and methods, and were calculated using GraphPad Prism 8.1.2 for Windows (GraphPad Software, San Diego, California USA, www.graphpad.com). Venn diagrams were generated using Venny 2.1.0 [70]. Gene ontology analyses were performed using STRING network [71] and g:Profiler [72] to highlight the biological processes modulated.

## Results

### Colorectal cancer cell lines have different intrinsic radio- and chemo-sensitivities

Radiation therapy combined with 5-FU-chemotherapy is the current standard of care treatment in the neoadjuvant setting for patients (TNM stages II and III) [64]. To assess the sensitivity to irradiation and 5-FU, six commonly used CRC cell lines, Caco-2/15, DLD-1, HCT-116, HT-29, SW480 and SW620 were treated with either treatment to measure the half maximal lethal dose (LD_50_) and half maximal inhibitory concentration (IC_50_), respectively. These cell lines had different genetic profiles and the mutations in key genes involved in CRC development have been characterized as well as the proteomic subtypes and the consensus molecular subtypes (CMS) (Table 1).

In order to determine the sensitivity to ionizing radiations, these cell lines were exposed to 2, 4, 6 and 8 Gy at D + 1 post-seeding and colony forming units (CFU) were counted after an 8-day or 12-day incubation (Fig. 1A; Fig. S1A). These results showed that these CRC cell lines had different intrinsic radio-sensitivities (Fig. 1B; Fig. S1B). The same experiments were performed to determine the 5-FU-sensitivity. These cell lines were subjected to increasing doses of 5-FU from 0.1 to 100 μM administered for 24 h at D + 1 post-seeding and CFU were counted after an 8-day or 12-day incubation (Fig. 1C; Fig. S1C). These results showed that these CRC cell lines also had different intrinsic chemo-sensitivities (Fig. 1D; Fig. S1D). Interestingly, the LD_50_ (irradiation treatment) and IC_50_ (5-FU treatment) values for all six cell lines were different and did not correlate (Table 1). For the rest of the experiments, three CRC cell lines were selected for further investigation: HCT-116, HT-29 and DLD-1 CRC cell lines, because they presented different genetic profiles and mutations, as well as different radio- and chemo-sensitivities (Fig. 1B, D; Table 1).

### Induction of a 5-FU-chemoresistance in colorectal cancer cell lines

**Fig. 2:**
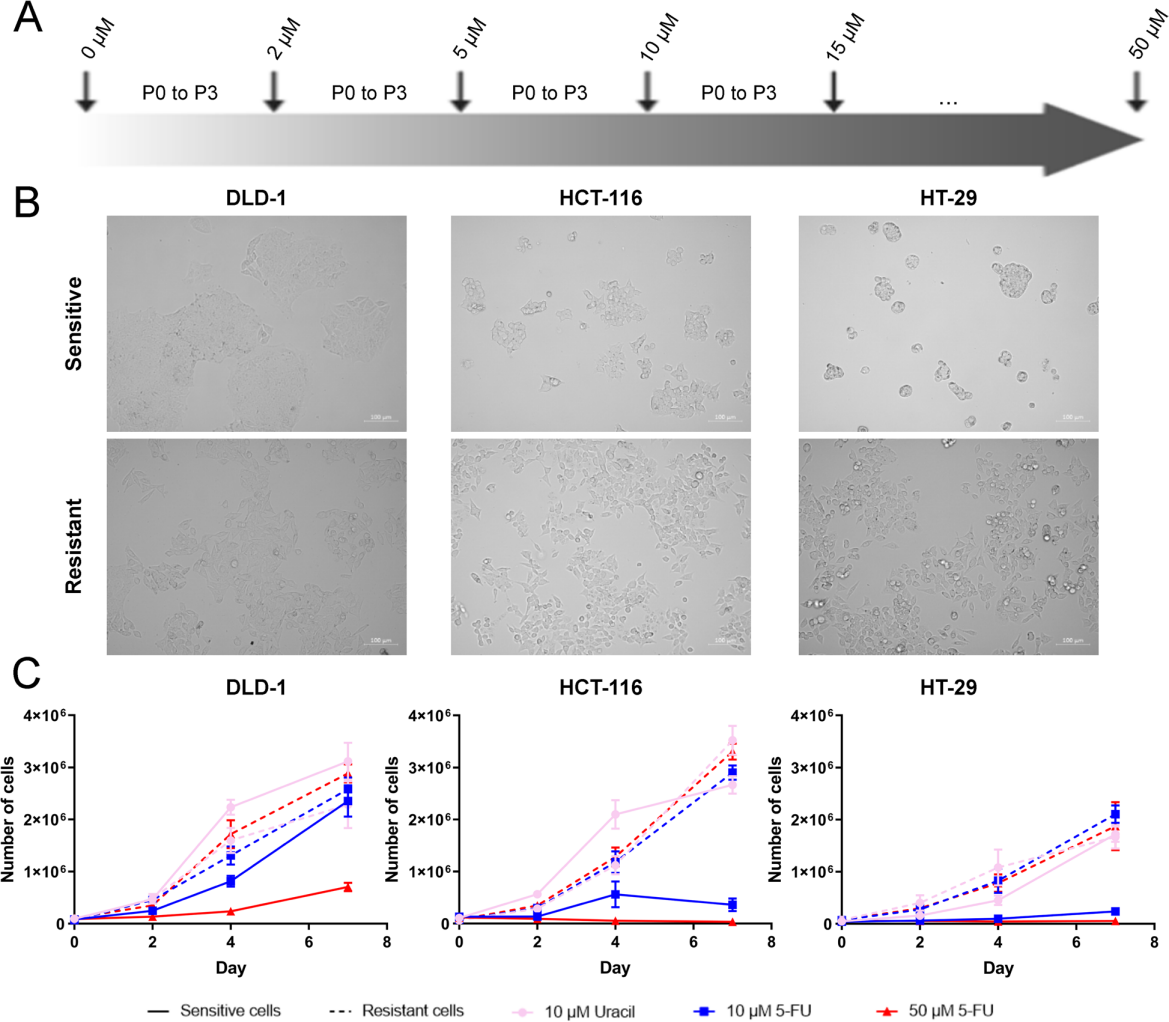
5-FU-resistant colorectal cancer cell lines normally grow in a high concentration of 5-fluorouracil. **A** Protocol for induction of a 5-FU-chemoresistance in CRC cell lines. DLD-1, HCT-116 and HT-29 were treated with increasing doses of 5-FU (culture medium is changed every two days). Cells underwent three passages in each dose of 5-FU. 5-FU treatments were started at 2 μM up to 50 μM by increasing the dose by 5 μM at each stage. **B** Morphological changes observed after induction of a 5-FU chemoresistance. Cells visualized under 10X magnification with Cell Discoverer 7 microscope. 5-FU-resistant DLD-1, HCT-116 and HT-29 CRC cell lines have a more fibroblastic appearance and demonstrate reduced intercellular contacts compare to the 5-FU-sensitive corresponding cell lines. Additionally, drug resistant cells extend pseudopodia. **C** Cell proliferation assays. 5-FU-sensitive (solid line) and -resistant (dotted line) DLD-1, HCT-116 and HT-29 CRC cell lines were seeded at D0 in six-well plates. They were treated at D + 2 for 24 h with 10 μM uracil (pink, circle) or 10 μM (blue, square) or 50 μM (red, triangle) 5-FU. They were counted at D + 2, D + 4 and D + 7 (three parallel samples in three independent experiments) in order to obtain these cell viability curves (GraphPad Prism 8.1.2). 5-FU: 5-fluorouracil; CRC: colorectal cancer

Chemoresistance commonly appears in cancer patients and represents a major obstacle to the success of their treatment. It can be intrinsic, but some patients develop it during the treatment. 5-FU-chemoresistance was induced in CRC cell lines in order to study this acquired chemoresistance by passaging cells with increasing concentrations of 5-FU up to 50 μM (Fig. 2A). Remarkably, morphological differences were observed between sensitive CRC cell lines and corresponding resistant cell lines. Resistant cell lines have a more fibroblastic appearance and demonstrate reduced intercellular contacts compared to the 5-FU-sensitive corresponding cell lines (Fig. 2B).

The proliferation of sensitive and resistant CRC cell lines in response to different doses of 5-FU (10 μM and 50 μM) administered for 24 h was measured for seven days to ensure that resistance was acquired in these cell lines (Fig. 2C). These graphs therefore confirmed that the cell lines were initially sensitive (Fig. 2C, solid lines, blue and red). After four days, sensitive cells treated with 10 μM 5-FU displayed the difference in sensitivity between the three cell lines previously observed with, in increasing order of 5-FU sensitivity, DLD-1 (IC_50_: 9.3 μM), HCT-116 (IC_50_: 4.5 μM) and then HT-29 (IC_50_: 2.7 μM) (Fig. 2C, solid lines, blue; Fig. 1C, D; Table 1). No difference was observed between treatments in resistant cell lines (Fig. 2C, dotted lines). These results validated the acquired chemoresistance in those cell lines.

### Transcriptomics and proteomics analysis of chemotherapy and radiotherapy in sensitive and resistant CRC cell lines

In order to study the changes in gene expression, quantitative proteomics and transcriptomics analyses were performed on these cell lines, comparing the resistant cells as well as the effect of chemo and radiotherapy.

**Fig. 3:**
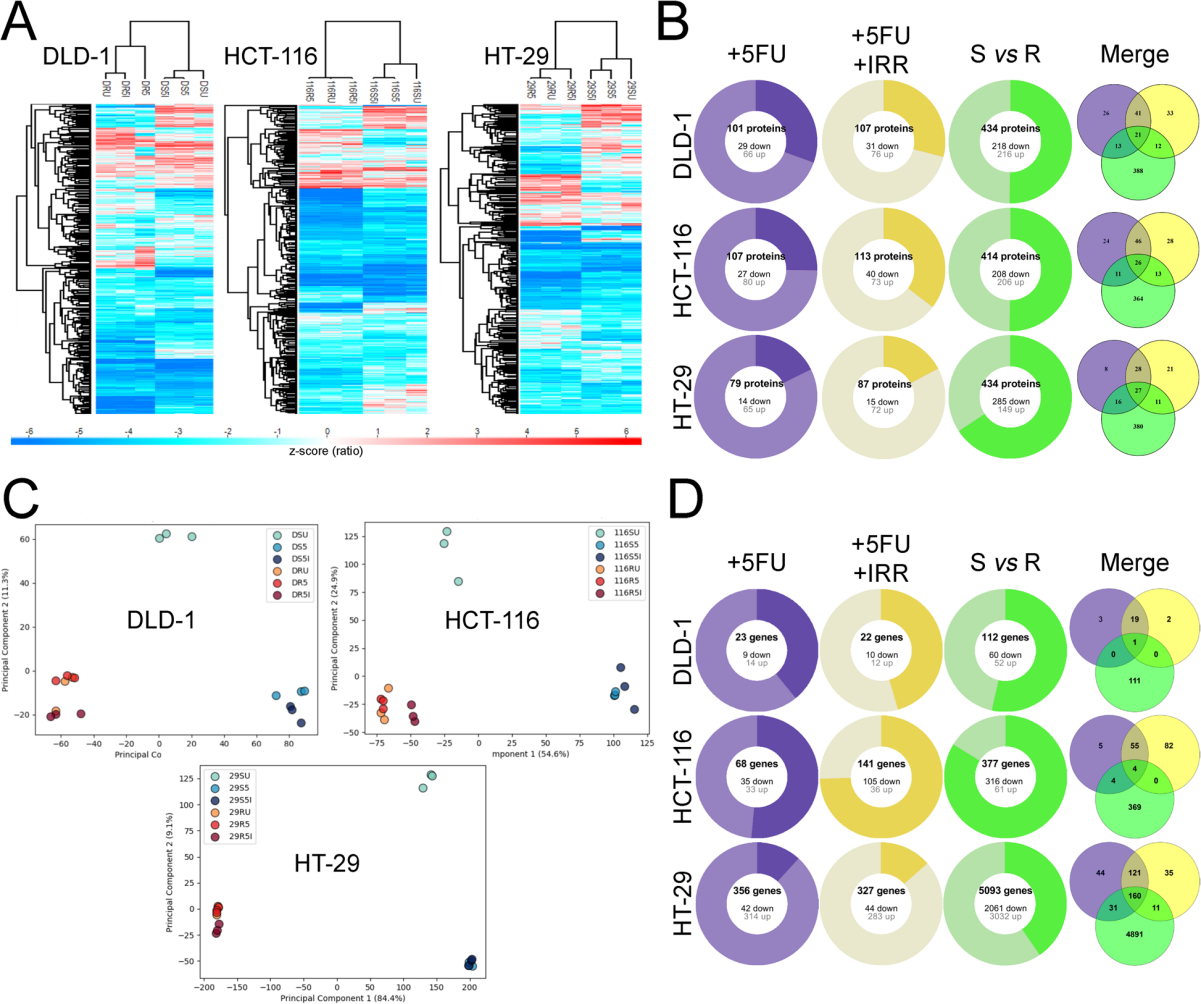
Transcriptomics and proteomics analysis of chemotherapy and radiotherapy in sensitive and resistant CRC cell lines. **A** Heat maps of the proteomics data from sensitive (S) and resistant (R) DLD-1 (D), HCT-116 (116) and HT-29 (29) CRC cell lines treated with uracil (U), 5-FU alone (5) or with irradiation (5I) (Perseus 1.6.12.0) [74]. **B** Representation of the number of significant up- (light) and down-regulated (dark) proteins for each CRC cell line after a treatment with 5-FU alone (+5FU, purple) or with irradiation (+5FU + IRR, yellow) compared to the control condition (uracil) and after the induction of a chemoresistance (S vs R, green). A minimum absolute z-score of 2 (calculated from the ratio treated/control) was used to determine significance. Venn diagrams (merge) highlight the common modulated proteins between the three conditions for each CRC cell line (Venny 2.1.0) [70]. **C** PCA analysis of the RNA seq data from sensitive (S) and resistant (R) DLD-1 (D), HCT-116 (116) and HT-29 (29) CRC cell lines treated with uracil (U), 5-FU alone (5) or with irradiation (5I). The biological triplicates for each sample are grouped by colors. The two main components PC1 and PC2 were used for two-dimensional visualization of the analysis. **D** Representation of the number of significant up- (light) and down-regulated (dark) transcripts for each CRC cell line after a treatment with 5-FU alone (+5FU, in purple) or with irradiation (+5FU + IRR, in yellow) compared to the control condition (uracil) and after the induction of a chemoresistance (S vs R, in green). A minimum absolute fold change of 2 combined with an adjusted p-value threshold ≤0.01 was used to determine significance. Venn diagrams (merge) highlight the common modulated transcripts between the three conditions for each CRC cell line (Venny 2.1.0) [70]

For the proteomics analysis, an approach using six-plex TMT was used to determine differences in protein expression. To achieve this, total proteins from each of the three cell lines (either sensitive or resistant to 5-FU) were treated under three different conditions (uracil, 5-FU or 5-FU + irradiation) then were extracted in triplicate. All those different conditions were then mixed differentially in order to allow comparison between the different cell lines and the different treatments, resulting in different 6-plex TMT mixes (Table S1). Hierarchical clustering analysis of the datasets was performed to determine the variance found between the proteins quantified for all cell lines and conditions (Fig. 3A). The treatment with 5-FU caused the most variance when compared to control, while treatment with irradiation exhibited very little additional effect. The results are viewed globally in Fig. 3B as circles whose respective share are proportional to the number of significantly modulated proteins. Significantly upregulated and downregulated proteins are presented as light and dark share of the circle, as well as the comparison between the sensitive and resistant cell lines (|z-score (ratio) ≥ 1.96|) (Fig. 3B, Tables S2.1–15, Tables S3.1–9).

Changes in gene expression at the transcriptional level in the different cell lines under different conditions was analyzed by RNAseq. To achieve this, total RNA from the three conditions (uracil, 5-FU or 5-FU + irradiation) for the three CRC cell lines either sensitive or following acquired resistance to 5-FU were extracted in biological triplicates. Sequencing of the 54 libraries was performed using NovaSeq 6000 (Illumina) using an S2 PE100 protocol. The number of reads varied between 56,377,534 and 240,114,519 for each sample. The readings were aligned to the human transcriptome constructed from the annotations from ENSEMBL (Homo sapiens version 98) (Table S4). Gene quantification analysis revealed that the annotation contained 59,369 genes, with 23,313 detected in at least one sample (using a minimal quantification of 1 TPM) (Table S5). The differential expression of transcripts and genes was obtained by comparing the specific data for each cell line compared to the control condition (Tables S6.1–15, Tables S7.1–9). Principal component analysis (PCA) of the datasets was performed to determine the variance found between the transcripts quantified for all cell lines and conditions as well as to validate the reproducibility of the triplicates for each experiment. This analysis confirmed the propinquity of each triplicate (Fig. 3C). The control condition (sensitive, uracil-treated) is annotated with the lightest shade, located on the top and center of the PC1 and PC2 components respectively. From these analyses, the treatment with 5-FU of sensitive cells caused the most variance when compared to control, while the treatment with irradiation in combination with 5-FU exhibited very little additional effect. Interestingly, treatment of resistant cells with 5-FU had very little effect on the overall transcriptome expression, suggesting that the gene expression responsible for the acquired resistance is already in place, and is not induced by the treatment of 5-FU (Fig. 3C). The differential expression was performed using the DESeq2 1.14.1 R package [69] and only those genes positively or negatively modulated by at least two-fold compared to the control, and with a maximum corrected p-value of 0.01, were considered. The results are viewed globally in Fig. 3D as circles whose respective sizes are proportional to the number of significantly modulated genes.

#### 5-FU differentially modulates proteome and transcriptome in the three sensitive colorectal cancer cell lines

**Fig. 4:**
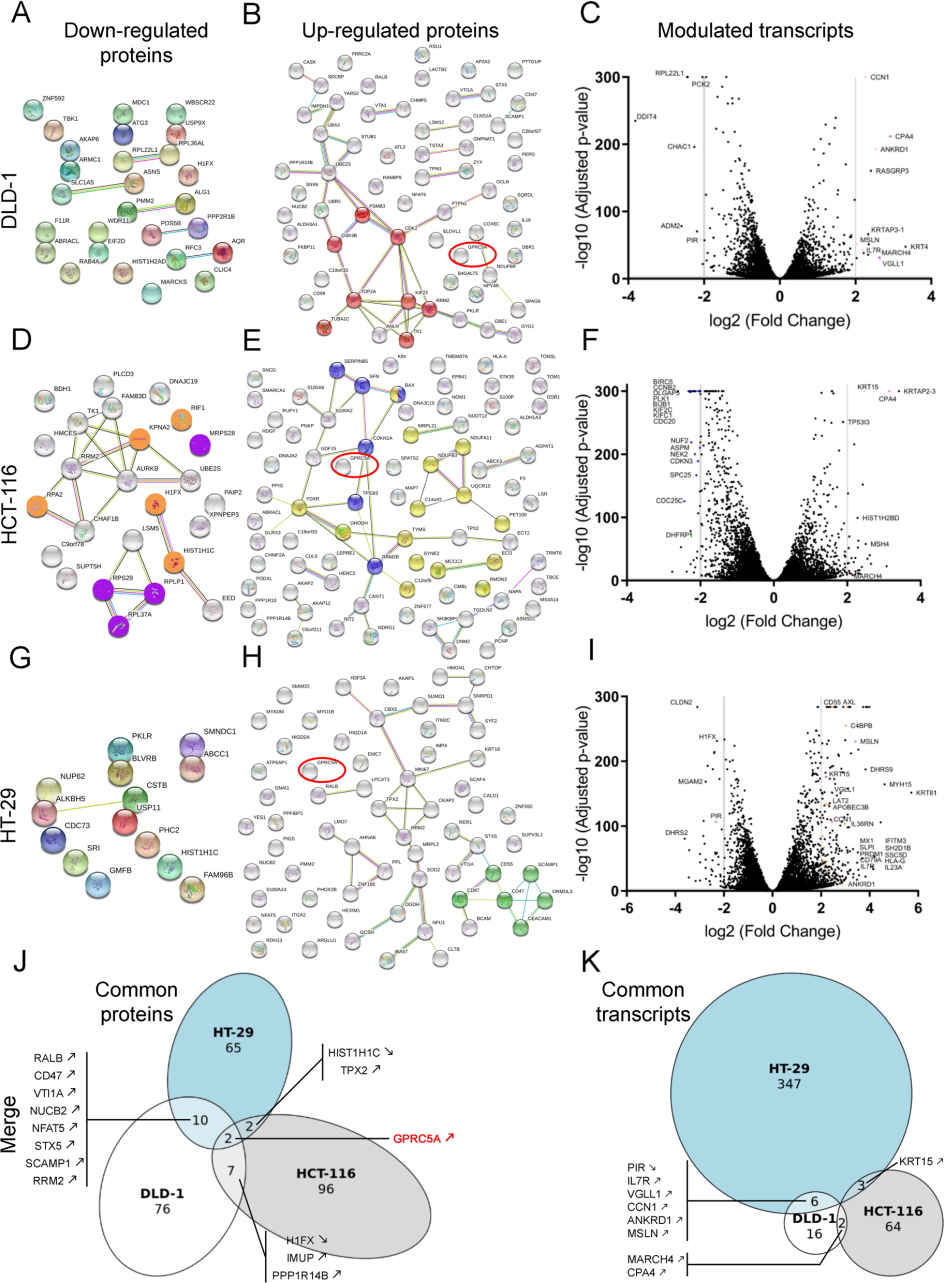
5-FU differentially modulates proteome and transcriptome in the three sensitive colorectal cancer cell lines. DLD-1 (**A-C**), HCT-116 (**D-F**) and HT-29 (**G-I**) CRC cell lines were subjected to a 10 μM 5-FU treatment during 24 h and their respective proteome and transcriptome were analyzed by TMT-6plex and RNA-sequencing in comparison with a 10 μM uracil treatment. Networks representing proteins with a statistically significant decreasing and increasing abundance in (**A-B**) DLD-1, (**D-E**) HCT-116 and (**G-H**) HT-29 CRC cell lines in response to 5-FU (STRING 11.0) [71] (|Z ≥ 1.96|). Volcano-plots representing transcripts differentially modulated in response to 5-FU in (**C**) DLD-1, (**F**) HCT-116 and (I) HT-29 CRC cell lines in response to 5-FU (GraphPad Prism 8.1.2) (|FC ≥ 2.0|). Venn diagrams representing modulated proteins (**J**) and transcripts (**K**) in common between the three CRC cell lines (eulerr.co) [76]. Up- or down-regulation of these proteins and transcripts in response to 5-FU are indicated by up or down arrows respectively. CRC: colorectal cancer; 5-FU: 5-fluorouracil

**Fig. 5:**
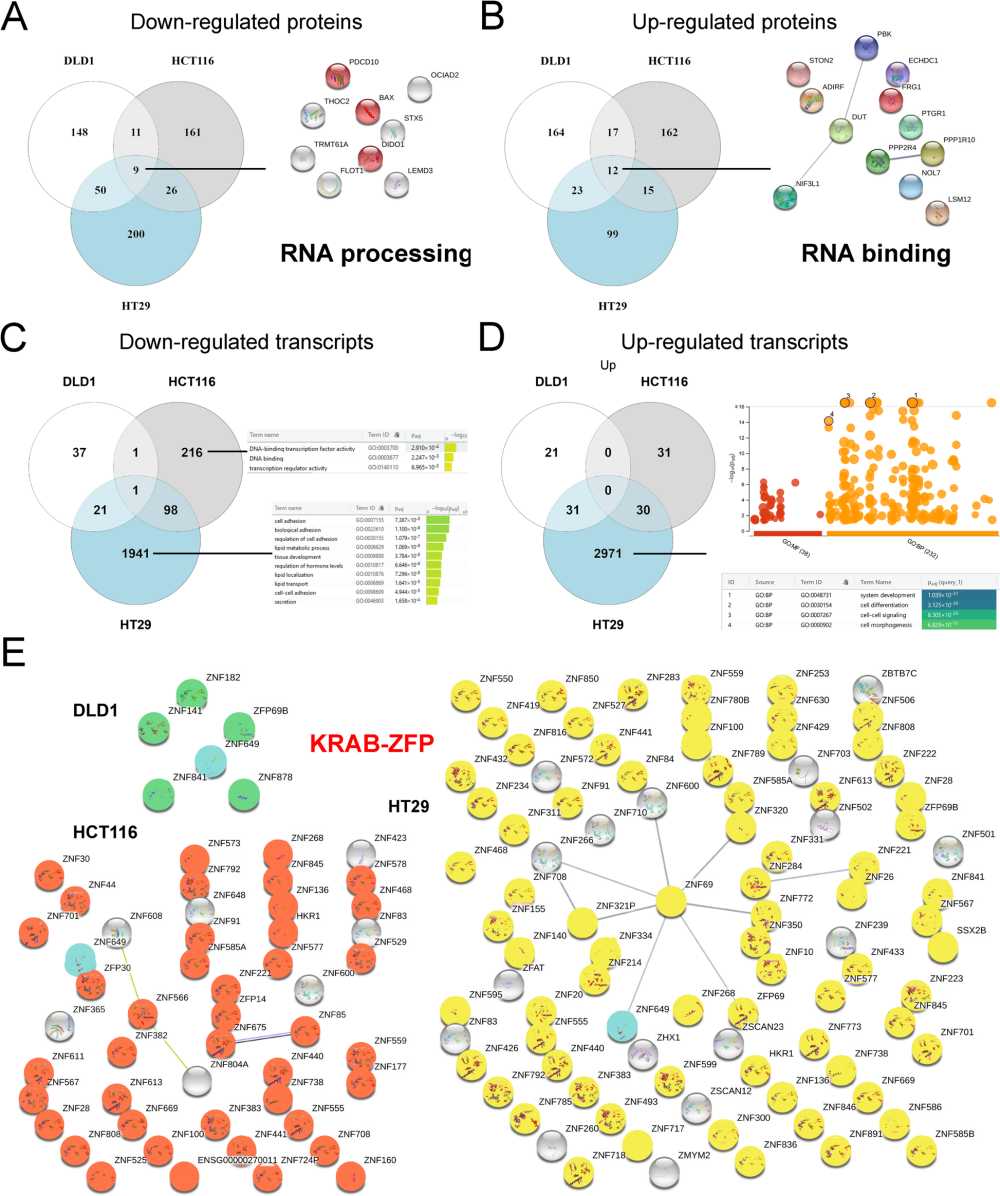
Induction of a 5-FU-resistance induced down-regulation of KRAB-ZFP in colorectal cancer cell lines. 5-FU-resistance was induced in DLD-1, HCT-116 and HT-29 CRC cell lines. Venn diagrams highlight the common modulated (**A-B**) proteins and (**C-D**) transcripts between the three CRC cell lines (Venny 2.1.0) [70]. STRING network [71] and g:Profiler tables and functional enrichment analysis [72] highlight the biological processes modulated after induction of a 5-FU-resistance. **E** STRING networks show genes corresponding to zinc finger proteins (ZFP) among the down-regulated transcripts after the induction of a chemoresistance in the three CRC cell lines. Highlighted genes (green for DLD-1, red for HCT-116 and yellow for HT-29) represent ZFP containing a Krüppel-associated box domain (KRAB-ZFP), a family of strong transcriptional repressors involved in regulation of many biological processes such as differentiation, metabolism and apoptosis. ZNF649 is highlighted in blue and is commonly down regulated in the three CRC cell lines

We then looked in more detail at the effect of 5-FU on the proteo-transcriptome of the three sensitive CRC cell lines (Fig. 4, Tables S2 and S3). The DLD-1 cell line, after a 10 μM 5-FU treatment, resulted in an increase in proteins involved in mitotic cell cycle (Reactome pathway: HSA-69278) (Fig. 4B, in red). Only RPL22L1 and ASNS are down-regulated both in terms of proteomics (Fig. 4A) and transcriptomics (Fig. 4C). No statistically significant biological process was observed (Tables S3.1, S7.1). Regarding the HCT-116 cell line, after 5-FU treatment, there was a decrease in proteins involved in *Regulation of DNA recombination* (GO:0000018) represented by H1FX, KPNA2, RIF1, HIST1H1C and RPA2 (Fig. 5D, in orange) and from *Ribosomal subunits* (GO:0044391) included RPS29, MRPS28, RPLP1 and RPL37A (Fig. 4D, in purple) (Tables S3.4). There was also an increase in proteins involved in *P53 signalling pathway* (KEGG:04115) [75] with SFN, BAX, SERPINB5, CDKN1A, RRM2B and TP53I3 (Fig. 4E, in blue) and from mitochondria (Fig. 4E, in yellow) including TYMS, the main enzyme targeted by 5-FU (Table S3.4). At the transcriptomic level, in response to 5-FU, we noted the strong downregulation of transcripts corresponding to proteins involved in the *Mitotic cell cycle process* (GO:1903047) (Table S7.4, Fig. 4F, in blue). Only TP53I3 is up-regulated both in terms of proteomics (Fig. 4E, in blue) and transcriptomics (Fig. 4F). Finally, for the HT-29 cell line, no statistically significant biological process was observed at the proteomic level (Fig. 4G, H, Tables S3.7) but some upregulated proteins from *Secretory granule membrane* (GO:0030667) can be observed (Fig. 4H, in green). At the transcriptional level, there was an increase in transcripts corresponding to secreted proteins and proteins involved in the *Immune response* (Fig. 4I, in orange). Only CD55 and ZNF185 are up-regulated both in terms of proteomics (Fig. 4H) and transcriptomics (Fig. 4I). We found very few commonly modulated proteins (Fig. 4J) or transcripts (Fig. 4K) between the three cell lines reflecting the very large heterogeneity in response previously described.

#### Induction of a 5-FU-resistance induced down-regulation of KRAB-ZFP in colorectal cancer cell lines

Finally, we were interested in proteins and transcripts modulated after the induction of 5-FU-chemoresistance in the three cell lines (Fig. 5). The induction of chemoresistance resulted in a much larger set of genes whose expression was differentially regulated. The number of genes that were up or down regulated was higher when compared to the effect of treatment with 5-FU on gene expression. Interestingly, the overlap between each cell line is very low, suggesting that even though they acquired resistance to 5-FU, the mechanisms involved appeared to be different (Fig. 4J, K).

At the proteome level, down-regulated proteins were found in the three cell lines such as PDCD10, OCIAD2, THOC2, STX5, TRMT61A, DIDO1, FLOT1, LEMD3 or even the pro-apoptotic protein BAX (Fig. 5A). Proteins commonly up-regulated were also found in the three lines, namely PBK, DUT, NIF3L1, STON2, ADIRF, ECHDC1, FRG1, PTGR1, NOL7, LSM12, PPP2R4 and PPP1R10 (Fig. 5B). The major observation is that the induction of chemoresistance to 5-FU largely affects the proteins involved in RNA metabolism (Fig. 5A, B; Tables S3.3, S3.6, S3.9).

**Fig. 6:**
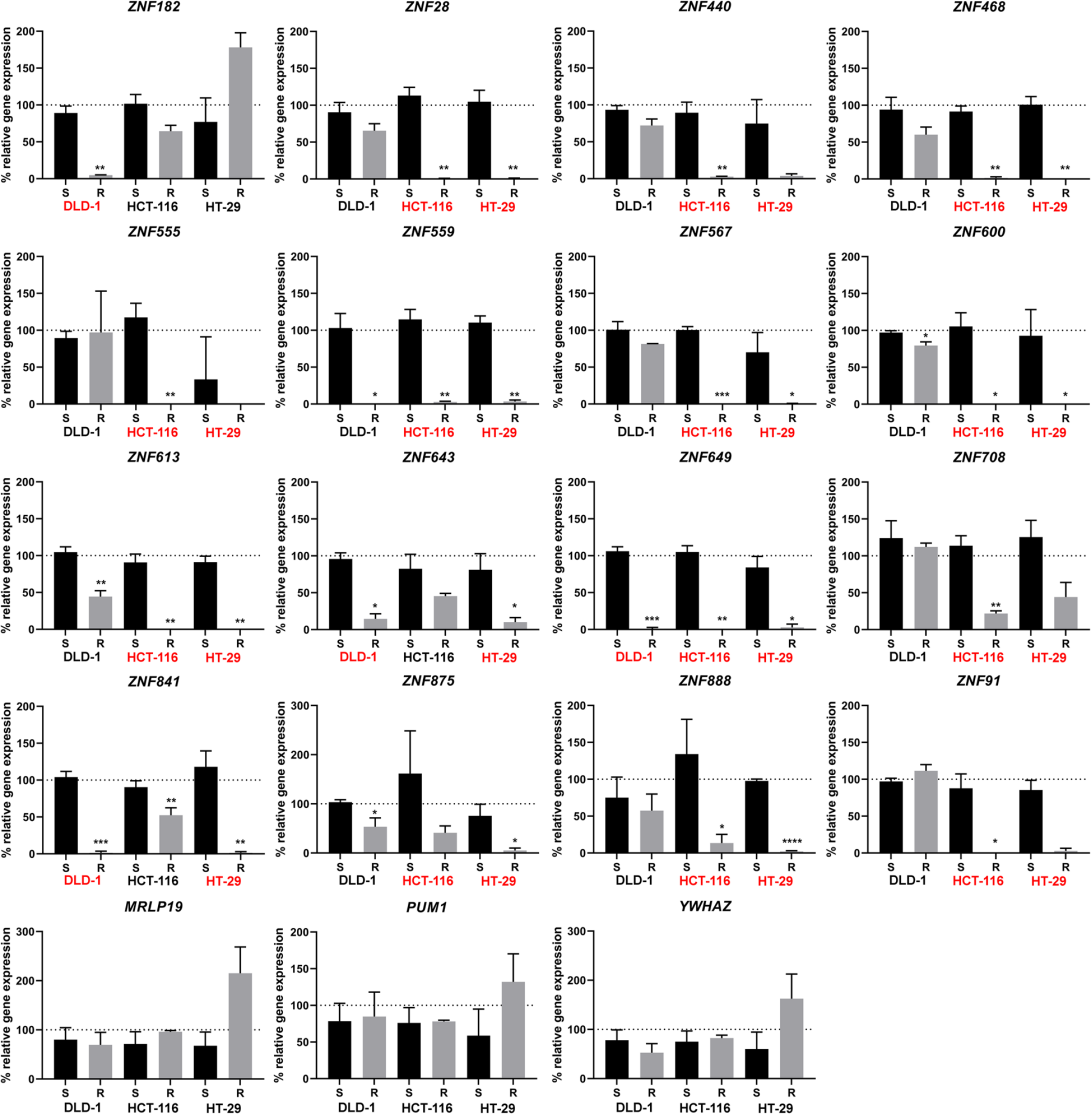
Quantitative RT-PCR assays on KRAB-ZFP proteins in sensitive and resistant cell lines. Total RNA was extracted from sensitive and resistant DLD-1, HCT-116 and HT-29 cell lines, and qRT-PCR was performed to quantify the relative amount of RNA using primers specific for the indicated genes. The cell lines indicated in red for each of the KRAB-ZFPs are the cell lines displaying downregulation of the mRNA as measured by RNAseq. MRLP19, PUM1 and YWHAZ were used as controls. Error bars indicate standard deviation. Asterisks represent significant P values (two-tailed Student’s t test) comparing the means between samples and their respective controls. *P < 0.05, **P < 0.01 and ***P < 0.001

**Fig. 7:**
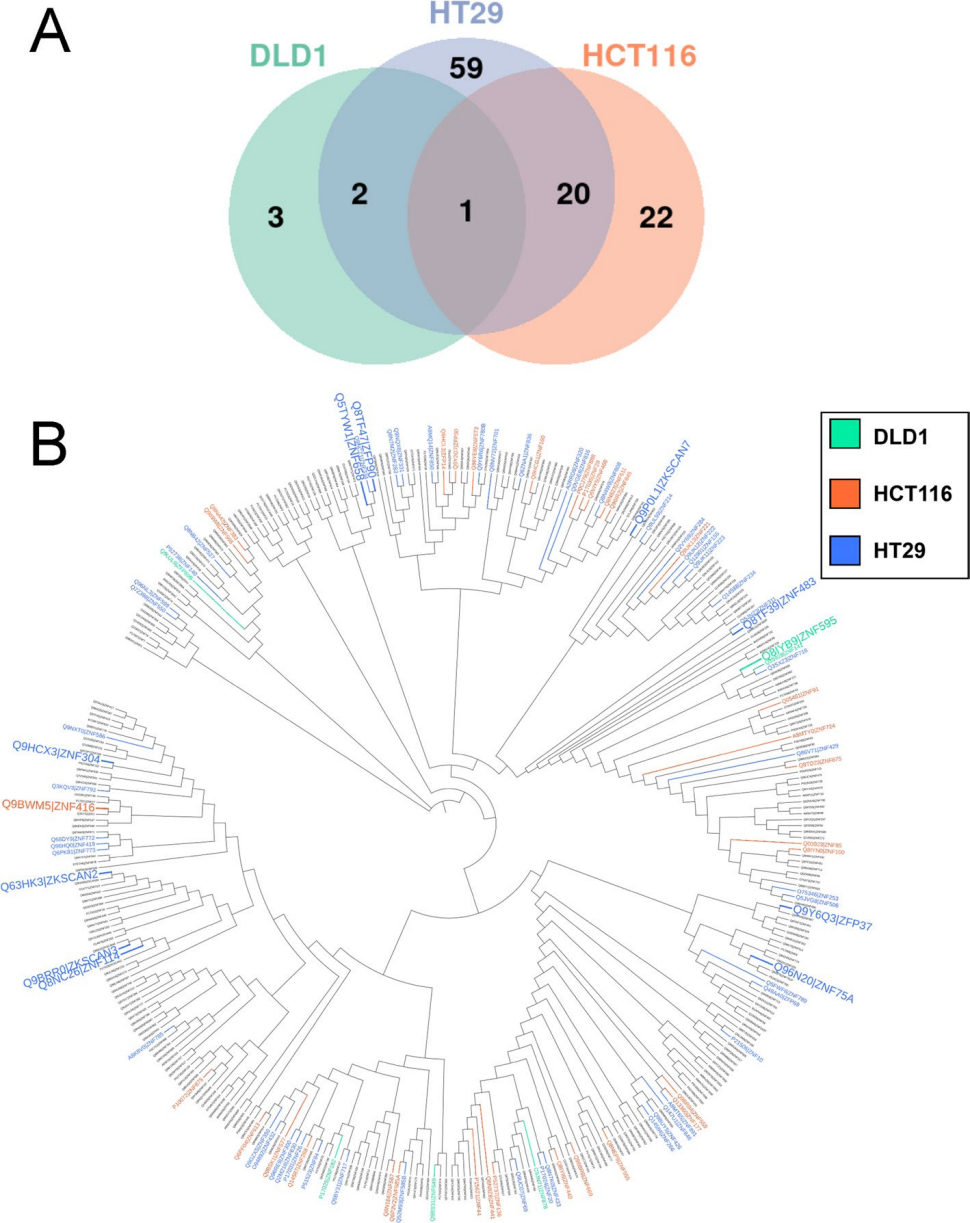
Phylogenetic analysis of regulated KRAB-ZFPs in 5-FU resistant cell lines. **A** Overlap of the KZFPs that shows changes in their expression in the resistant DLD-1, HCT-116 and HT-29 cells. **B** A phylogenetic analysis of the 361 KZFPs in human, with the different genes that are either downregulated (small fonts) in each of the cell lines (green = DLD-1, red = HCT-116 and blue = HT-29), or upregulated (large fonts)

At the transcriptome level, there are few transcripts common to the three cell lines after induction of resistance to 5-FU (Fig. 5C, D) and therefore no significant common biological processes were identified (Tables S7.3, S7.6, S7.9). However, the most significant molecular function that was found to be downregulated was transcription repression and included Krüppel-associated box (KRAB) domain-containing zinc-finger proteins (KZFP) (Fig. 5E) including ZNF649, the only common down-regulated transcript between the three cell lines (Fig. 5E, in blue). To confirm the observation that KZFPs were downregulated, quantitative RT-PCR assays in sensitive and resistant cell lines were performed using a subset of the downregulated KZFPs. Total RNA was extracted from sensitive and resistant DLD-1, HCT-116 and HT-29 cell lines, and qRT-PCR was performed to quantify the relative amount of RNA using primers specific for the indicated genes (Fig. 6). The cell lines indicated in red for each of the KZFPs are the cell lines displaying downregulation of the mRNA as measured by RNAseq, which overall shows a very strong correlation between the two types of experiments (Fig. 6). This experiment confirmed that ZNF649 is indeed downregulated in all three cell lines, as well as ZNF559. Moreover, it confirmed that DLD-1 cells have fewer KZFPs that are downregulated as compared with HCT-116 and HT-29 (Fig. 5E). To determine whether the different KZFPs that are downregulated in each of the resistant cell lines have common characteristics (Fig. 7A), a phylogenetic tree analysis was performed based on the similarities between each of the 361 known KZFPs in human (Fig. 7B). The KZFPs that were downregulated were found throughout the different genes, further confirming that it is not the downregulation of a specific subfamily or potential similar target genes which results in 5-FU resistance. The function of most KZFPs is unknown, but several have been demonstrated to be involved in transcriptional repression. Interestingly, there are only two KZFPs that are repressed in all three resistant cell lines, and also very few that were found in two different cell lines. Altogether, our data suggest that it is not a specific role of some KZFPs that are involved in the resistance to 5-FU, but perhaps the downregulation of transcriptional repression allows cells to grow in high concentration of 5-FU.

## Discussion

CRC is a multifactorial and very heterogeneous cancer, which has given rise to multiple classifications over time [5, 77]. Indeed, CRC can be classified according to its hereditary nature or not, the type of carcinogenesis pathway used (CIN or MSI) or even different combinations of parameters: the colon cancer subtype (CCS) system [8], the colorectal cancer assigner (CRCA) system [4], the colon cancer molecular subtype (CCMS) system [9], the colorectal cancer intrinsic subtype (CRCIS) system [10] and the colorectal cancer subtyping consortium (CRCSC) [78]. These numerous classifications highlight the heterogeneity of CRC and therefore the importance of personalized medicine for better management and better response of patients to treatments. For example, the treatment of locally advanced rectal cancer (LARC, TNM stages II-III) in North America consists of radiotherapy combined with chemotherapy with 5-fluorouracil (5-FU) in neoadjuvant condition (NRCT). Many studies report rates of non-responders around 30%, which is a considerable percentage for a treatment used in common practice. Herein, we studied several colorectal cancer cell lines (DLD-1, HCT-116 and HT-29) with different genetic characteristics (Table 1) to identify and understand the mechanisms associated with intrinsic and acquired radio- and 5-FU-chemoresistance using proteo-transcriptomic analyses.

First, we showed that six colorectal cancer cell lines had different intrinsic 5-FU-chemosensitivities, from the most sensitive to the most resistant: Caco-2/15 (1.4 μM), SW480 (1.4 μM), HT-29 (2.7 μM), HCT-116 (4.5 μM), SW620 (5.2 μM) and DLD-1 (9.3 μM) (Fig. 2B, D, Table 1). Bracht et *al.* found substantially the same order in terms of sensitivity for these cell lines as our study (the IC_50_ obtained were different but the techniques for determining the latter were also different) [79]. The only major difference consists of the HT-29 cell line which had a very low sensitivity to 5-FU (IC_50_ = 14.05 μM in their study, against 2.7 μM in ours). The HT-29 cell line has an MSS phenotype, which does not exhibit microsatellite instability. This phenotype has repeatedly been associated with normal expression of genes involved in mismatch repair (MMR) and with a good response to 5-FU treatment [79, 80]. This observation supports the higher resistance of the HCT-116 and DLD-1 cell lines, which, for their part, have a MSI+ phenotype. We also observed different intrinsic radio-sensitivities with, from the most sensitive to the most resistant: HCT-116 (1.7 Gy), SW620 (2.6 Gy), SW480 (2.8 Gy), DLD-1 (2.9 Gy), Caco-2/15 (4.5 Gy) et HT-29 (4.6 Gy) (Fig. 2A, C, Table 1), which is also supported by the literature. Indeed, Tippayamontri et *al.* had defined an LD_50_ of 2.3 ± 0.98 Gy for HCT-116 cell line [81] which is very close to the value observed in our study. In addition, it has been shown that the DLD1 and SW620 cell lines were much more radiosensitive than the HT-29 and Caco-2/15 cell lines [82, 83] and that HCT-116 cell line was much more radiosensitive than the DLD1 [84] and HT-29 [85] cell lines. It has also been shown that in response to radiation, there was activation of the PI3K/AKT/mTOR signaling pathway [86]. This pathway is known to be involved in cell survival and growth. It is also implicated in the tumorigenesis of CRC [87] and can play a role in radio-resistance. This pathway can also be aberrantly activated by mutations in the *K-RAS* gene, a mutation carried by almost 50% of patients with CRC [88] and by HCT-116 et DLD-1 cell lines (*K-RAS* G13D) (Table 1), but this mutation cannot alone explain the level of intrinsic radio-sensitivity in our cell lines. The notable difference between these two cell lines is the presence of the *TP53* Ser241Phe mutation in the DLD-1 cell line. This mutation results in a defective P53 signalling pathway [89, 90] and could partly explain the differences in intrinsic radio- and chemosensitivities between these two cell lines.

Secondly, we induced a 5-FU-chemoresistance in the DLD-1, HCT-116 and HT-29 lines in order to study the mechanisms involved in acquired chemoresistance, a phenomenon which is observed in many patients during their treatment. To achieve resistance in cell lines, we followed the protocol described in Fig. 2 up to the point where they grow normally in a concentration of 50 μM of 5-FU. Interestingly, and similarly to Ahn *et al.*’ study, morphological changes were observed between the sensitive lines and the corresponding resistant CRC cell lines (Fig. 2B) [91]. We then subjected the sensitive and 5-FU resistant CRC cell lines to different treatments supposed to “mimic” chemotherapy and radiotherapy. The effects of chemotherapy alone (24-h 10 μM 5-FU treatment) or in combination with radiotherapy (24-h 10 μM 5-FU treatment + irradiation with the corresponding LD_50_) on the proteome and transcriptome were evaluated. 5-FU, by its mechanism of action, interferes with replication but also with DNA repair. It is also incorporated into RNA and DNA, causing damages. The result of radiotherapy is to induce cell death by causing DNA damages, and the cellular responses are staggered over time: early recognition of damage (a few seconds), signaling and repair of lesions (a few tens of minutes), induction or repression of genes (a few hours) then cell death (a few hours to a few days). It has been estimated that an irradiation dose of 1 Gy causes on average 1000 single-strand breaks (SSB) per cell and 40 double strand breaks (DSB) [92]. The synergistic effect of 5-FU with irradiation is caused by its ability to redistribute cells in the S phase and to deplete the nucleotide pool, which decreases the cell’s ability to repair DNA.

We performed TMT-6plex and RNA sequencing experiments to assess proteomic and transcriptomic changes, respectively, in sensitive and resistant CRC cell lines in response to chemotherapy and radiation treatments. As expected, the first observation was that the number of proteins and transcripts modulated in response to 5-FU treatment was proportional to the level of intrinsic sensitivity to 5-FU of the three CRC cell lines, which was also more obvious at the transcriptional level) (Fig. 3). At the proteome level, fewer significantly modulated proteins were detected than in the Marin-Vicente *et al.* study [50] but the mass spectrometry techniques and CRC cell lines used were different (label-free quantification (LFQ) *versus* TMT; RKO cell line much more 5-FU sensitive than HCT-116, DLD-1 and HT-29 cell lines [79]). On the other hand, 5-FU being a radio-sensitizer, we expected to have a large increase in proteins and transcripts modulated with the addition of irradiation, but this increase was lower than expected. Finally, the number of proteins and transcripts modulated between the sensitive cell lines and the corresponding resistant cell lines was also compared. At the transcriptome level, there was clearly an absence of significantly modulated transcripts in resistant cell lines even with irradiation. We know that one of the effects of 5-FU is to interfere with the maturation of RNA and it is therefore logical that we observe a strong transcriptomic impact. We assume that the impact at the proteome level would also be stronger after recovery times greater than 48 h.

We then looked in more detail at the effect of 5-FU on the proteo-transcriptome of CRC cell lines (Fig. 4). The DLD-1 cell line, after a 10 μM 5-FU treatment, resulted in an increase in proteins involved in mitotic cell cycle (Fig. 4B, in red). This observation was not found at the transcriptional level (Fig. 4C) and led us to believe that in response to 5-FU, the DLD-1 cell line, which represents the cell line with the lowest intrinsic chemosensitivity to 5-FU, would promote the repair of misincorporations. A more in-depth study on the mechanisms of reparation like mismatch repair (MMR) or base excision repair (BER) could be carried out to validate this. Regarding the HCT-116 cell line, after 5-FU treatment, there was an increase in proteins involved in P53 signalling pathway especially the pro-apoptotic protein BAX and TP53I3 (Fig. 4E, in blue), also increased at the transcriptomic level (Fig. 4F). TP53I3, also known as PIG3 (P53 Inducible Gene 3), is induced by P53 to activate apoptosis. PIG3 is also required to activate the DNA damage response pathway. Indeed, Lee et al. have shown that after DNA damage, PIG3 co-localizes with the γ-H2AX foci and that in the absence of PIG3, there is a significant reduction in the phosphorylation of CHK1, CHK2 and H2AX, proteins involved in the DDR [93]. At the transcriptomic level, in response to 5-FU, we noted the downregulation of many transcripts corresponding to proteins involved in the cell cycle and mitosis including CCNB2 or CDC25C (Fig. 4F, in blue). At the proteomic level, we also noted the decrease in the abundance of proteins involved in the regulation of DNA recombination (Fig. 4D, in orange). These observations lead us to believe that in response to 5-FU, there would be a cell cycle arrest in the HCT116 cell line and that in the absence of repair of the damage (which could be explained by the MSI+ status of the HCT-116 cell line among others), it would favor death by P53-dependent apoptosis. This hypothesis could be verified by caspase tests, for example. In the HT-29 cell line, in response to 5-FU, there was an increase in proteins and transcripts involved in immune response (Fig. 4H, in green, 4I, in orange) which might suggest a senescence-associated secretory phenotype (SASP). Even more interesting, the HT-29 cell line is known to have the BRAF V600E mutation and a link has been shown between SASP and that mutation [94–97]. We had found very few elements modulated in common between the three cell lines (Fig. 4J, K) reflecting the very large heterogeneity in response previously described. Nevertheless, GPCR5A, an orphan G protein–coupled receptor seems to be decreased in the three cell lines in response to 5-FU (Fig. 4B, E, H, red circles). This receptor would operate as a negative modulator of EGFR signaling. It has already been identified as a lung tumor suppressor [98] and an oncogene in the pancreatic context [99].

Finally, we were interested in proteins and transcripts modulated after the induction of 5-FU-chemoresistance in the three cell lines (Fig. 5). At the protein level, RNA metabolism seems to be very affected which is logical since the main target of 5-FU is the inhibition of the maturation of RNA in the cell [27, 33, 34, 36, 100–102]. The most significant effect observed in response to the induction of a 5-FU-chemoresistance and common to the three CRC cell lines was the decrease in transcripts of genes involved in transcriptional repression, which mainly corresponded to ZFPs containing a KRAB domain (Krüppel-associated box). Through this domain, these ZFPs recruit complexes involved in deacetylation in the regions surrounding their DNA or RNA binding site, making them powerful transcriptional repressors. Nevertheless, the function of most KRAB-ZFPs is unknown. The only common transcript between the three CRC cell lines corresponds to the protein ZNF649 which has almost never been studied in particular [103–105]. Moreover, there were also very few KZFPs that were found downregulated in two cell lines. We therefore suggest that it is the general alleviation of transcriptional repression, which would be responsible for the resistance to 5-FU, rather than the effect of specific KRAB-ZFPs. It will be interesting to validate the role of ZKFPsthrough overexpression in resistant cells, as well as through downregulation in sensitive cells to confirm whether their regulation is directly responsible for the resistance to 5-FU, or instead a consequence.

## Supplementary Information

**Additional file 1.**

**Additional file 2.**

**Additional file 3.**

**Additional file 4.**

## Data Availability

**Proteomics data deposition.** The mass spectrometry proteomics data have been deposited to the ProteomeXchange Consortium [106] via the proteomics identification database (PRIDE) partner repository [107] with the dataset identifier PXD020000. **Transcriptomics data deposition.** The RNAseq dataset has been deposited into the NCBI’s Gene Expression Omnibus (GEO; https://www.ncbi.nlm.nih.gov/geo/) [108] and are accessible through GEO Series accession number GSE153412. The entire pipeline used for sequence alignment described below is available at http://gitlabscottgroup.med.usherbrooke.ca/berd2710/colorectal-cancer-resistance/tree/master.
