# Supplementary figures and images for: Downregulation of KRAB zinc finger proteins in 5-fluorouracil resistant colorectal cancer cells
Source: BMC Cancer. 2022 Apr 4;22:363. doi: 10.1186/s12885-022-09417-3 (PMC8981854; doi:10.1186/s12885-022-09417-3)

Figure S1

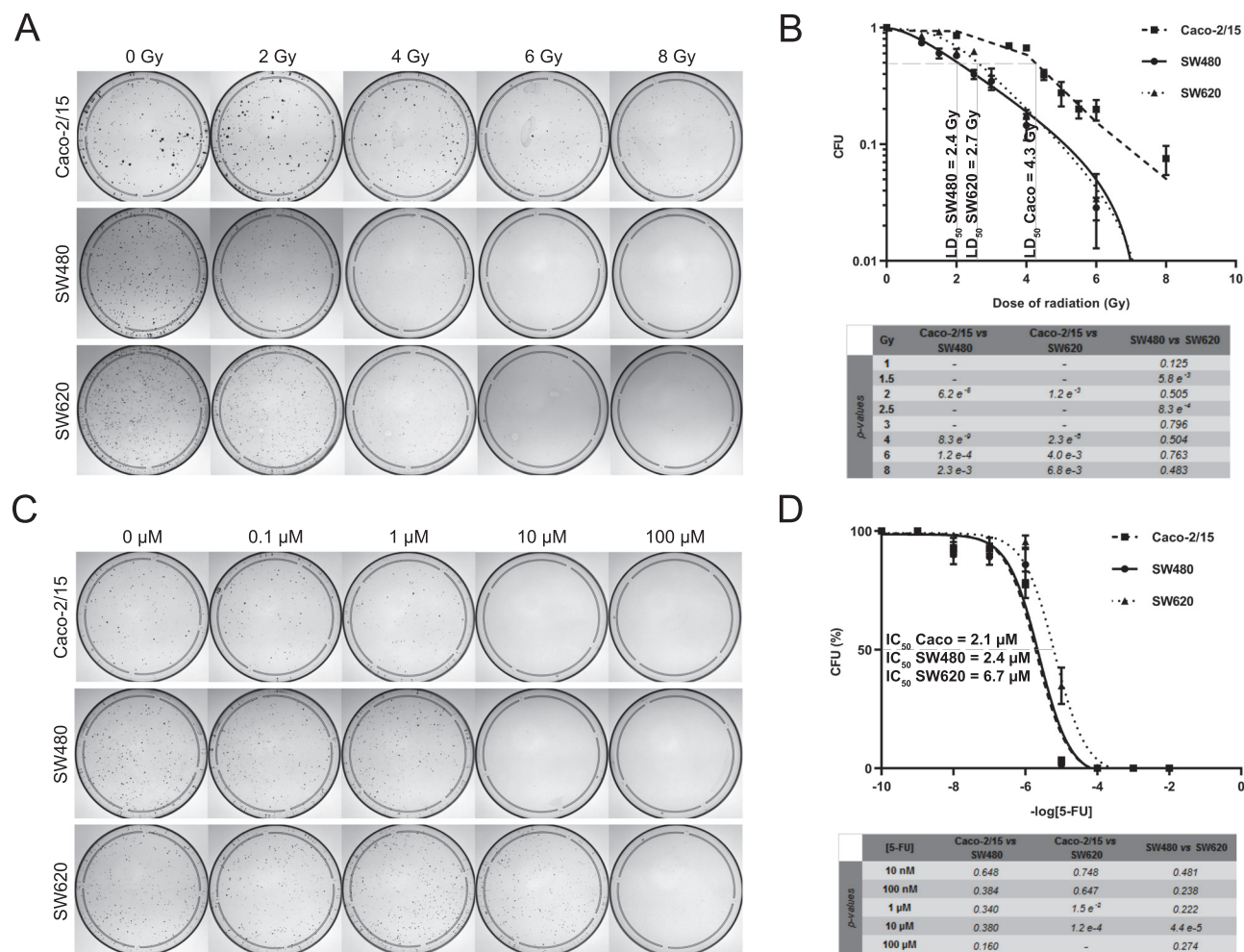

Supplement: Supplementary file 2 — Additional file 2. [file 12885_2022_9417_MOESM2_ESM.pdf]

Figure S2

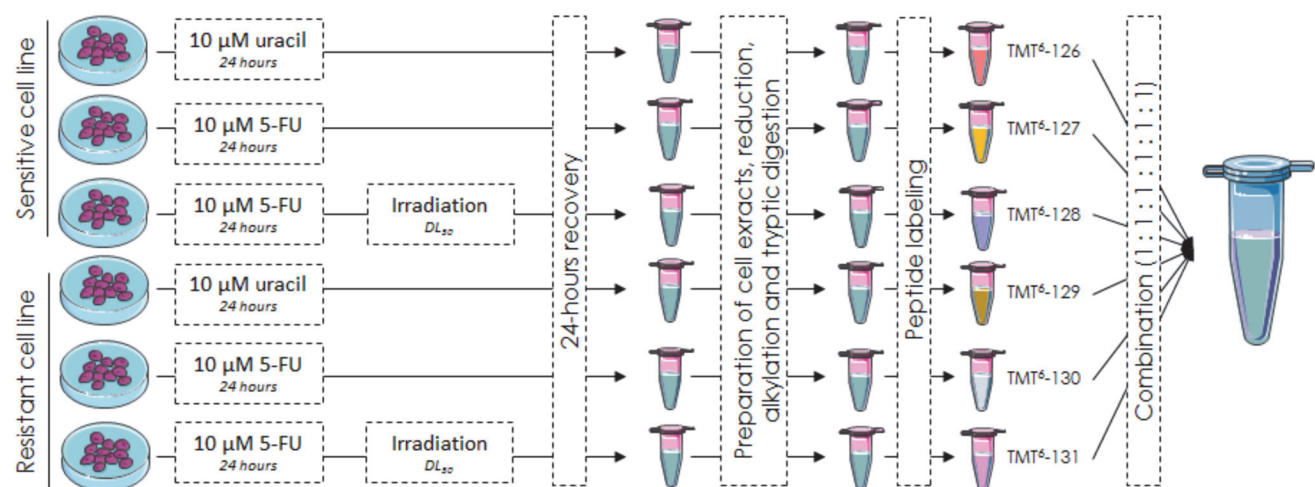

Supplement: Supplementary file 3 — Additional file 3. [file 12885_2022_9417_MOESM3_ESM.pdf]
