# Supplementary material for: Downregulation of KRAB zinc finger proteins in 5-fluorouracil resistant colorectal cancer cells
Source: BMC Cancer. 2022 Apr 4;22:363. doi: 10.1186/s12885-022-09417-3 (PMC8981854; doi:10.1186/s12885-022-09417-3)
Supplement: Supplementary file 4 — Additional file 4. [file 12885_2022_9417_MOESM4_ESM.docx]

**Supplementary materials and methods**

***Clonogenic assays.*** Cell colony formation assays were performed to determine the half maximal lethal dose (LD_50_) and the half maximal inhibitory concentration (IC_50_) of ionizing radiations and 5-FU, respectively, in the Caco-2/15, DLD-1, HCT-116, HT-29, SW480 and SW620 CRC cell lines. For each cell line, 1000 cells were seeded in 100 mm Petri dish for 24 hours. To mimic the radiotherapy, cells were irradiated with single doses of 2, 4, 6 or 8 Gy (three parallel samples in three independent experiments for each radiation dose for each cell line). To mimic the 5-FU chemotherapy, medium was supplemented with 0.1, 1, 10 or 100 μM 5-FU for 24 hours (three parallel samples in three independent experiments for each 5-FU dose for each cell line). After treatment, medium was changed, and CRC cell lines were incubated for 8 days (DLD-1, HCT-116 and SW620) or 12 days (Caco-2/15, HT-29 and SW480). CRC cell lines were fixed and stained with 0.1% crystal violet and colonies containing more than around fifty cells (CFU) were counted using ImageJ (<https://imagej.nih.gov/ij/>) (93). Dose-response curves and IC_50_ and LD50 were obtained with GraphPad Prism 8.1.2 for Windows (GraphPad Software, San Diego, California USA, [www.graphpad.com](http://www.graphpad.com)).

***Immunoblotting.*** Cells were lysed in 1 mL RIPA buffer (50 mM Tris-HCl pH 7.5, 0.1% SDS, 150 mM NaCl, 5 mM EDTA pH 8.0, 1% NP40, 0.5% sodium deoxycholate, 50 mM NaF, 1 mM PMSF and complete EDTA protease inhibitor). The cell lysates were centrifuged for 5 minutes at 4°C and sonicated at 25% amplitude for 30 seconds. The amounts of protein in the supernatants were measured with Thermo Scientific^TM^ Bicinchoninic acid (BCA) protein assay kit (#23225). Western blots were performed as described here (94). Briefly, an amount of 25 μg of proteins was loaded on Novex™ WedgeWell™ 4 to 12%, Tris-Glycine, 1.0 mm, Mini Protein Gel, 12-well. After a semi-dry transfer onto a nitrocellulose membrane, blocking was performed in 5% milk. After three washes, membranes were incubated overnight with the primary antibody diluted in primary antibody buffer (1% BSA, 10 mM Tris pH 7.4, 150 mM NaCl, 0.01% NaN3). After three washes, membranes were incubated 2 hours in secondary antibody diluted in secondary antibody buffer (2% BSA, 10 mM Tris pH 7.4, 150 mM NaCl, 1% milk). Membranes were then revealed with Clarity Western ECL substrate (BioRad, #1705061) in a Chemidoc XRS+ system (BioRad). The following primary antibodies were used: anti-GAPDH (rabbit monoclonal, #5174S, 1:50000), anti-phospho-CHK1(Ser345) (rabbit monoclonal, #2348, 1:1000), anti-phospho-CHK2(Thr68) (rabbit monoclonal, #2197, 1:1000), anti-phospho-P53(Ser15) (mouse monoclonal, #9286, 1:1000) and anti-phospho-histone H2A.X(Ser139) (rabbit monoclonal, #9718, 1:1000). The following secondary antibodies were used: anti-rabbit IgG-HRP (goat, #7074S, 1:10000) and anti-mouse IgG-HRP (horse, #7072, 1:10000).

***Quantitative RT-PCR assays***

Total RNA from sensitive or resistant DLD-1, HCT-116 and HT-29 cells was extracted using the RNeasy RNA isolation kit (#74104). The RNA concentrations were measured by NanoDrop and RNA qualities were evaluated on 1.2% agarose gel. The 36 samples were sent to the RNomics Platform of the Université de Sherbrooke, and the oligonucleotides used as primers are listed in Supplementary Table S8. Reverse transcription was done using 10U of Transcriptor reverse transcriptase, 20 U of RNaseOUT (Invitrogen), 3.2 µg of random hexamers, 1 µM of dNTPs mix, 1× Transcriptor RT reaction buffer and 0.2–2 µg of total RNA. PCR was done using 0.2 U of Platinium Taq, 0.6 µM of primers, 1.5 mM of MgCl2, 10 ng of cDNA template, 1× of PCR buffer and 200 µM of dNTPs mix. PCR reactions were performed on thermocyclers GeneAmp PCR System 9700 (Thermo Scientific-Invitrogen). A first cycle of 15 min at 95 °C was followed by 35 cycles of 30 s at 94 °C, 30 s at 55 °C, and 1 min at 72 °C. The reaction was ended with the extension step of 10 min at 72 °C. Visualization and analysis of amplified products were done using automated chip-based microcapillary electrophoresis on Labchip GX Touch HT instruments (Perkin Elmer).
